# Internet-Based CBT for Depression with and without Telephone Tracking in a National Helpline: Randomised Controlled Trial

**DOI:** 10.1371/journal.pone.0028099

**Published:** 2011-11-30

**Authors:** Louise Farrer, Helen Christensen, Kathleen M. Griffiths, Andrew Mackinnon

**Affiliations:** 1 Centre for Mental Health Research, The Australian National University, Canberra, Australian Capital Territory, Australia; 2 Biostatistics Unit, Orygen Youth Health Research Centre, University of Melbourne, Melbourne, Victoria, Australia; University of Muenster, Germany

## Abstract

**Background:**

Telephone helplines are frequently and repeatedly used by individuals with chronic mental health problems and web interventions may be an effective tool for reducing depression in this population.

**Aim:**

To evaluate the effectiveness of a 6 week, web-based cognitive behaviour therapy (CBT) intervention with and without proactive weekly telephone tracking in the reduction of depression in callers to a helpline service.

**Method:**

155 callers to a national helpline service with moderate to high psychological distress were recruited and randomised to receive either Internet CBT plus weekly telephone follow-up; Internet CBT only; weekly telephone follow-up only; or treatment as usual.

**Results:**

Depression was lower in participants in the web intervention conditions both with and without telephone tracking compared to the treatment as usual condition both at post intervention and at 6 month follow-up. Telephone tracking provided by a lay telephone counsellor did not confer any additional advantage in terms of symptom reduction or adherence.

**Conclusions:**

A web-based CBT program is effective both with and without telephone tracking for reducing depression in callers to a national helpline.

**Trial Registration:**

Controlled-Trials.com ISRCTN93903959

## Introduction

Telephone helplines provide 24 hour, non-directive crisis and counselling services to large numbers of callers in the community using a volunteer workforce. In 2009, major helplines in the United Kingdom received over 4 million calls [1]. In Australia, over 850,000 calls are answered annually by the large telecounselling providers, with 20% of callers referred by healthcare providers. Although designed primarily to provide immediate crisis intervention, these services are frequently used by callers experiencing chronic mental health problems. Over 40% of callers phone the service 20 times or more, 78% of callers experience depressive symptoms, and the prevalence of anxiety in this group is between 2 to 6 times that of the population [2], [3], [4]. In addition to providing non-directive counselling, telephone helpline agencies refer callers to mainstream mental health services [5]. However, mental health services are often difficult to access because of cost, structured consultation times, waiting list length, and location. In addition, mainstream mental health services may not inspire trust.

An alternative to referral to another agency is to provide low cost, evidence-based, high quality mental health interventions within the helpline service itself. The telephone and the Internet have been used separately to effectively treat physical and mental health problems [6], [7], [8], [9]. Cognitive behaviour therapy (CBT) offered through the telephone has been shown to significantly reduced depression [10]. A range of software applications available on the web have also delivered effective CBT to depressed samples [11], reduced depression in school settings [12] and in general practice environments [13], [14], either with or without support from mental health professionals, paraprofessionals or teachers [11]. Based on reviews of the evidence, the provision of support or tracking (ongoing contact with users during an intervention) has been proposed to increase adherence to treatment, prevent dropout, and improve efficacy [6]. Reviews of Internet-based interventions for depression have shown that treatments delivered with therapist support show larger between-group effect sizes than unsupported treatments [6], [8]. However, few studies have compared supported and unsupported interventions in the same trial [11] and so the issue of whether support adds unequivocal benefit has not yet been resolved. In addition, efficacy may vary depending on the type of support provided (e.g. automated versus non-automated, professional versus non-professional). Like therapist supported treatments, there is evidence to suggest that treatments involving assistance from a non-professional are more effective than control conditions for depression [7], [15], [16], [17]. Evidence from Titov and colleagues suggests that treatments supported by professionals and non-professionals are equally effective in the treatment of depression [18]. However, to-date, no studies have directly compared the relative efficacy of an Internet-based self-help treatment monitored by non-professionals (such as volunteer telephone counsellors) with a non-guided treatment.

One method of implementing a low cost, high quality intervention within a helpline service may be to provide callers with proven web-based programs for anxiety and depression in combination with telephone tracking by a telecounsellor. Volunteer counsellors constitute a ready made workforce to refer and/or guide callers, making it possible to use the existing call centre structure to deliver immediate, cost-effective, evidence-based help. However, it is important to establish the effectiveness of such a service and to what extent if at all counsellor guidance is likely to improve the outcomes of the service.

To our knowledge, no trials have evaluated the effectiveness of web based interventions within existing helpline or call centre services. Our study aimed to evaluate the effectiveness of a web-based CBT program delivered within a helpline, and to examine the benefit of weekly telephone contact (tracking) on efficacy, adherence and dropout. Over a period of 6 weeks, participants were randomised to one of four conditions: *web only*, where participants received web-based automated depression psychoeducation combined with 5 modules of CBT, *web with tracking*, where callers received the web intervention and were phoned weekly by telephone counsellor to monitor their completion of the intervention, *tracking only*, where callers were telephoned weekly but were not provided with the web intervention, and the *control* condition, where participants received neither the web intervention nor weekly telephone calls. In all four conditions, participants were able to contact and receive usual care from the helpline service, if required.

We hypothesised that (a) the delivery of a web-based CBT program would be more effective than the control condition; (b) that the combination of tracking with the web intervention would be superior to the other three conditions, and (c) that the addition of tracking to the web intervention (web with tracking) would be superior to delivery of the web only condition in terms of effectiveness and adherence.

## Methods

The protocol for this trial and supporting CONSORT checklist are available as supporting information; see Checklist S1 and Protocol S1.

### Ethics statement

Ethical approval for the trial was granted by the Australian National University Human Research Ethics Committee (Protocol no. 2007/12).

### Participants

Callers to Lifeline's 24 hour telephone counselling service in four major Australian cities were invited to participate in the trial by a telephone counsellor either during or at the conclusion of a counselling call, between July 2007 and January 2009. Callers who were considered by the telephone counsellor to be suicidal or experiencing high levels of distress were excluded from receiving a recruitment invitation. Callers willing to receive further information about the trial provided contact information and were later telephoned and screened for eligibility by the trial coordinator. Callers were eligible for inclusion if they spoke English, had Internet access for at least half an hour a week, were aged 18 years or older, and scored 22 or above on the 10 item Kessler Psychological Distress Scale (K10) [19]. The exclusion criteria were a current or previous diagnosis of schizophrenia or bipolar disorder, current treatment with CBT, and significant reading impairment, and these were assessed directly using single item questions. Ineligible participants were offered brochures containing information about the online interventions.

### Design and procedures

Following screening, informed written consent was obtained from participants and baseline data were collected through a self report questionnaire mailed to participants. A block randomisation procedure with stratification based on sex, site of recruitment and severity of psychological distress at screening was used. Allocation of participants to trial conditions was conducted independently by a research assistant not otherwise involved with the trial. Following randomisation, all participants were contacted by telephone and mailed the relevant materials for their allocated condition.

### Intervention and trial conditions

#### Web only

The web only intervention delivered online psychoeducation (in Week 1, provided by BluePages: bluepages.anu.edu.au) combined with CBT (in weeks 2–6, provided by MoodGYM: moodgym.anu.edu.au). Both BluePages and MoodGYM have been shown to reduce depressive symptoms in community users [15]. A printed manual containing week by week instructions for accessing the web programs (via a login) was sent to participants by mail at the start of the trial.

BluePages is a freely accessible, psychoeducational website that contains information and resources related to depression. The website includes information about the symptoms of depression, ratings of the effectiveness of various depression treatments, and links to available help and resources. The MoodGYM program is a free to end-user, online CBT program for depression. The program is divided into 5 modules designed to be completed sequentially. Each module focuses on a particular aspect of CBT, such as cognitive restructuring, the relationship between thoughts and feelings, behavioural activation, relaxation, and problem solving. Each MoodGYM module contains written information, animations, interactive exercises and quizzes.

#### Web with tracking

In the web with tracking condition, participants completed the web intervention and also received a weekly 10 minute telephone call from a telephone counsellor, with the call addressing any issues associated with the participants' use of the online programs.

#### Tracking only

In the tracking only condition, participants received a weekly 10 minute phone call from a telephone counsellor. The calls focussed on various environmental and lifestyle factors associated with depression. No advice, therapy or counselling was offered in either of the tracking conditions.

#### Control condition

In the control condition, participants received neither tracking nor the web intervention.

Participants in all 4 conditions were invited to use the helpline, which provided usual emergency or support services if required.

### Measures

Data were obtained by self report questionnaires mailed to participants at baseline, post intervention, and 6 months post intervention.

#### Screening and demographic variables

Previous history of depression, panic, social phobia and specific phobia were assessed at screening. Sex, age, years of education, relationship status, and employment status were assessed at baseline.

#### Depression

Depression symptoms were measured by the Center for Epidemiologic Studies Depression Scale [20] (CES-D), a widely used, 16 item scale with strong reliability and validity [20], [21], [22], [23], [24], [25], with a range of scores of 0 to 60. The CES-D was used as a continuous variable to examine symptom severity, and was also dichotomised into depression ‘cases’ and ‘non-cases’ in order to map the results into clinically meaningful outcomes. Depression caseness was measured by a score of 16 or above on the CES-D [20]. Studies have estimated reasonable sensitivity (84%–93%) and specificity (69%–74%) of the CES-D using this cutoff [26], [27].

#### Dropout

Dropout was measured by questionnaire completion at post intervention and 6 months.

#### Adherence

Program adherence was measured by number of visits to the BluePages psychoeducation site and by the number of MoodGYM program modules completed (ranging from 0 to 5).

### Power

The total target sample size was 400 with 100 participants assigned to each intervention arm. This would enable the detection of differences of means of less than 0.3 standard deviation (SD) units comparing treatment arms, with 80% power and ά = .05, conservatively assuming a correlation of 0.5 between measurements on different occasions. This sample size was motivated by the desire to evaluate the augmentation of the web intervention with tracking, a difference which was not expected to be as large as for the intervention itself. However, the rate of recruitment prompted reconsideration of the target sample size. The achieved sample size of 155 participants maintained power above 80% to detect differences of means of less than 0.5 standard deviation (SD) units in planned comparisons between individual treatment arms. Power calculations were undertaken using G*Power 3.1.0 [28].

### Statistical analysis

Data were analysed using SPSS release 18.0.1 for Windows [29]. A significance level of .05 was used for all outcome variables. The primary outcome variable was analysed on an intention to treat (ITT) basis using mixed models repeated measures ANOVA, with measurement occasion as a within groups factor and intervention condition as a between groups factor. Within person variation was modelled using an unstructured covariance matrix. Widely used methods of dealing with missing observations due to drop out such as ‘last observation carried forward’, treat the substituted values as real observations and make uncertain assumptions about the mechanism of missingness. Mixed modelling allows the use of all available data for each participant to yield unbiased estimates of effects under the assumption that missingness is either completely at random (MCAR) or at random conditional on observed data (MAR) [30]. Planned contrasts were used to compare intervention and control groups at post intervention and 6 month follow up. While multiple tests are reported post intervention and at 6 month follow-up, adjustments to *p*-values were not undertaken in the primary analyses due to the a priori nature of the comparisons involved and to ensure comparability of results with simpler two-arm trials.

Effect sizes (Hedges' *g*) were calculated by dividing the mean difference between conditions at post intervention and 6 month-follow up by the pooled standard deviation of the groups, which was computed using the method proposed by Hedges [31]. Confidence intervals for the effect sizes were calculated using the method proposed by Kelley [32]. Logistic and linear regressions examined differences between conditions on all baseline variables, and logistic regressions were used to assess the effect of the intervention on participant dropout. Logistic regressions were also used to investigate predictors of missingness (failure to complete a questionnaire) post intervention and at 6 month follow-up. The number needed to treat (NNT) was calculated for participants above caseness criteria at baseline. NNT is an index of the effectiveness of an intervention reflecting the average number of participants meeting criteria for a disorder who need to be treated in order to result in one person remitting from the condition. Confidence intervals for NNT may include infinity and extend to regions which imply that, comparatively, participants are harmed by an intervention as indexed by an analogous number needed to be harmed (NNH) value [33]. Confidence intervals were estimated using the method proposed by Bender [34]. Data inspection prior to analysis suggested that two cases in the web with tracking condition had aberrant patterns of change, each having moderate CES-D scores at baseline and extremely severe scores post intervention. In one case, this was able to be linked to the diagnosis of a life threatening illness. Due to distortion of the variance and violation of the assumption of normality [35], these participants were deleted for the primary analyses. However, additional analyses were conducted with these cases included.

## Results

### Participation rates and sample characteristics

**Figure 1:**
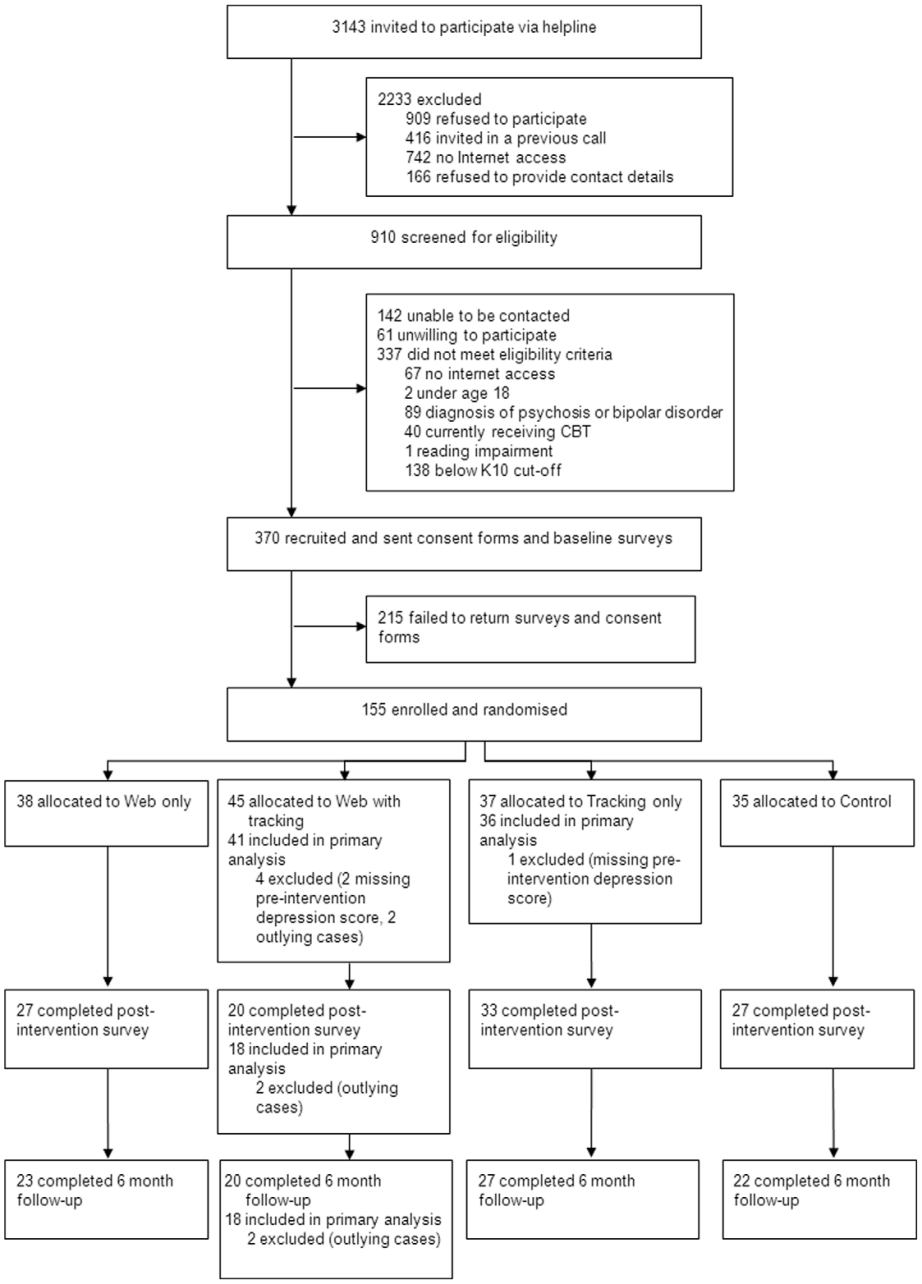
Flow of participants through the trial.

3143 callers were invited to be screened for participation in the trial during the recruitment period (Figure 1). Of these, 910 (28.9%) agreed to be screened for eligibility. 142 (15.6%) were unable to be contacted, 61 (6.7%) were later unwilling to participate, and 337 (37.0%) did not meet eligibility criteria. The most common reasons for ineligibility were a score of less than 22 on the K10 (138; 40.9%), a diagnosis of schizophrenia or bipolar disorder (89; 26.4%), or no Internet access (67; 19.9%). 370 people were eligible for inclusion in the trial, and of these, 155 completed informed consent procedures and baseline assessments, and were randomised to trial conditions, resulting in a 42% acceptance rate. 107 (69%) returned post intervention surveys, and 92 (59%) completed the 6 month follow up.

There were no significant differences across trial conditions on any baseline variables (Table 1). Trial condition and number of MoodGYM modules completed were significant predictors of missingness (failure to complete a questionnaire) at post intervention. The odds of missingness at post intervention were greater for participants in the Internet plus tracking condition, relative to participants in the control condition (odds ratio 6.46, 95% CI: 2.01 to 20.77, *p* = .002). Those who completed more MoodGYM modules had reduced odds of missingness at post intervention (odds ratio 0.49, 95% CI: 0.35 to 0.69, *p*<.001). At 6 month follow-up, those who completed more MoodGYM modules also had reduced odds of missingness (odds ratio 0.56, 95% CI: 0.41 to 0.75, *p*<.001). Pre intervention psychological distress, sex, age, pre intervention depression symptoms, pre intervention anxiety symptoms and whether participants received their “preferred” trial condition did not predict missingness at post intervention or 6 month follow-up.

**Table 1:** Baseline characteristics of randomised participants.

	Condition			
	Web only (n = 38)	Web with tracking (n = 45)	Tracking only (n = 37)	Control (n = 35)
Sex (female), n (%)	33 (86)	37 (82)	29 (78)	28 (80)
Age (years), mean (SD)	37.5 (12.0)	41.7 (12.1)	43.4 (12.6)	43.7 (12.3)
Years spent in education*, mean (SD)	13.3 (2.7)	14.0 (2.7)	13.1 (2.6)	13.4 (2.7)
Relationship status, n (%):				
Married/cohabiting	10 (26)	11 (24)	14 (38)	8 (23)
Divorced/separated/widowed	11 (29)	21 (47)	16 (43)	16 (46)
Never married	17 (45)	13 (29)	7 (18)	11 (31)
Employment status*, n (%):				
Employed full time	8 (21)	9 (20)	10 (27)	9 (26)
Employed part time	11 (29)	13 (29)	4 (11)	8 (23)
Not in labour force	19 (50)	22 (49)	23 (62)	18 (51)
History of previous depression*, n (%)	35 (92)	41 (91)	33 (89)	35 (100)
Experience of panic attacks in last 4 weeks*, n (%)	26 (68)	33 (73)	27 (73)	29 (83)
History of social phobia*, n (%)	19 (50)	29 (64)	24 (65)	21 (60)
History of specific phobia*, n (%)	8 (21)	7 (16)	5 (14)	5 (14)
K-10 score, mean (SD)	32.4 (6.3)	32.4 (5.6)	32.4 (5.9)	34.0 (5.5)
CES-D score*, mean (SD)	35.0 (10.8)	34.4 (10.2)	37.6 (10.7)	38.6 (8.8)
Caseness (score on CES-D ≥16)*, n (%)	36 (95)	42 (93)	35 (95)	35 (100)

Not all participants completed all questions.

K-10 = Kessler 10 Psychological Distress Scale.

CES-D = Centre for Epidemiological Studies - Depression Scale.

*Education (n = 150); Employment (n = 154); Depression history (n = 154); Panic attacks (n = 153); Social phobia (n = 151); Specific phobia (n = 152); Baseline CES-D (n = 152); Caseness (n = 152).

### Depression symptoms

**Figure 2:**
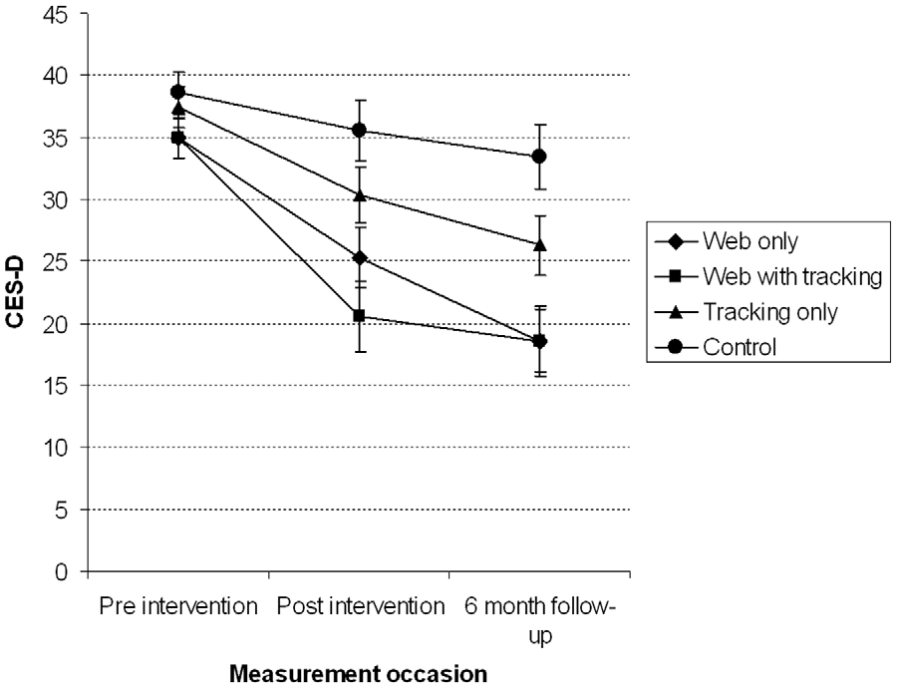
Estimated marginal means and standard errors (±1 SE) for depression (CES-D) symptoms.

A significant occasion by condition interaction was found for depression symptoms, *F*(6,100.5) = 2.97, *p* = .01), indicating different patterns of change between conditions across measurement occasion. Observed means for all conditions at baseline, post intervention and 6 month follow-up are shown in Table 2. Figure 2 displays mean depression symptoms over time for each condition. Planned contrasts tested the differences in change across conditions from baseline to post intervention and 6 month follow up (Table 3). Compared to the control condition, decline in depression symptoms from baseline to post intervention was greater for participants in the web only and web with tracking conditions. Decline in symptoms did not differ significantly between the web only and web with tracking conditions from baseline to post intervention. Symptoms for participants in the tracking only condition fell between the control and the web only conditions, and did not differ significantly from either. This pattern of results was repeated in all contrasts comparing change from baseline to the 6 month follow-up. Effect sizes for the difference between the intervention and control conditions at post intervention and 6 month follow-up are presented in Table 4. Post intervention, large effect sizes were observed between the web only and the control condition (*g* = 0.76) and the web with tracking and the control condition (*g* = 1.04). The sizes of these effects increased at 6 month follow-up (*g* = 1.19 and 1.26, respectively). Moderate effects were observed when the web intervention conditions were compared to the tracking only condition at post intervention and at 6 month follow-up.

**Table 2:** Observed means (SD) and caseness (%) on the CES-D for each trial condition at pre intervention, post intervention and six month follow up.

	Measurement occasion								
	Pre intervention			Post intervention			6 month follow-up		
	*n*	*Mean (SD)*	*Caseness (%)*	*n*	*Mean (SD)*	*Caseness (%)*	*n*	*Mean (SD)*	*Caseness (%)*
CES-D									
Web only	*38*	35.0 (10.8)	36 (94.7)	*27*	24.4 (13.6)	19 (70.4)	*23*	18.0 (13.0)	10 (43.5)
Web with tracking	*41*	34.9 (10.1)	40 (97.6)	*18*	21.0 (12.4)	12 (66.7)	*18*	18.4 (10.4)	9 (50.0)
Tracking only	*36*	37.6 (10.7)	35 (97.2)	*33*	29.6 (13.2)	29 (87.9)	*27*	26.0 (13.7)	18 (66.6)
Control	*35*	38.6 (8.8)	35 (100.0)	*27*	35.1 (13.9)	25 (92.6)	*22*	34.2 (13.5)	20 (90.9)

**Table 3:** Contrast estimates and significance tests for contrasts between conditions at post intervention and 6 month follow up.

	Pre intervention to post intervention			
		Control	Tracking only	Web only
Web with tracking	Estimate	**−11.32**	**−7.33**	4.68
	95% CI	**−18.04–−4.60**	**−13.83–−0.83**	−2.03–11.40
	Test value	**t(109.2) = −3.34, p = 0.00**	**t(109.5) = −2.23, p = 0.03** *	t(109.2) = 1.38, p = 0.17
Web only	Estimate	**−6.63**	−2.64	
	95% CI	**−12.74–−0.53**	−8.50–3.22	
	Test value	**t(105.8) = −2.16, p = 0.03** *	t(105.7) = −0.89, p = 0.37	
Tracking only	Estimate	−3.99		
	95% CI	−9.86–1.88		
	Test value	t(105.4) = −1.35, p = 0.18		

	Pre intervention to 6 month follow up			
Web with tracking	Estimate	**−11.23**	−5.23	−0.03
	95% CI	**−18.72–−3.73**	−12.45–2.00	−7.46–7.41
	Test value	**t(100.5) = −2.97, p = 0.00**	t(100.7) = −1.43, p = 0.16	t(100.0) = −0.01, p = 1.00
Web only	Estimate	**−11.25**	−5.25	
	95% CI	**−18.31–−4.20**	−12.02–1.52	
	Test value	**t(100.3) = −3.16, p = 0.00**	t(100.4) = −1.54, p = 0.13	
Tracking only	Estimate	−6.00		
	95% CI	−12.84–0.84		
	Test value	t(100.7) = −1.74, p = 0.09		

Significant findings are bolded.

*These contrasts are not significant (p<.05) under adjustment (Bonferroni correction) for multiple comparisons.

**Table 4:** Effect sizes (Hedges' g) and 95% confidence intervals for differences between the intervention and control conditions at each measurement occasion.

	Measurement occasion			
	Post intervention		6 month follow up	
	*g*	95% CI	*g*	95% CI
Web only vs control	0.76	0.21–1.31	1.19	0.55–1.83
Web with tracking vs control	1.04	0.40–1.67	1.26	0.57–1.94
Web only vs Tracking only	0.38	0.14–0.89	0.58	0.01–1.15
Web with tracking vs control	0.65	0.06–1.24	0.60	0.02–1.20

Analyses were repeated with the two outlying cases included. The findings were consistent with those reported above but resulted in slightly attenuated effect sizes and reduced or quasi significant test results. For example, pre to post intervention change remained significantly different comparing web with tracking to control (*t*(112.6) = −2.09, *p* = .039) and nearly so for web only condition compared to the control condition (*t*(109.0) = −1.98, *p* = .051). At 6 months, comparable outcomes were (*t*(107.7) = −1.72, *p* = .089) and (*t*(107.7) = −2.83, *p* = 0.006) respectively.

### Clinical caseness

Table 2 shows numbers and percentages of participants meeting clinical caseness criteria in each condition at each measurement occasion. Four participants were not classified as clinical cases pre intervention. Of those for whom post intervention data were available, 92.6% of those in the control condition continued to meet clinical caseness, compared to 70.4% in the web only and 66.7% in the web with tracking condition. A univariate logistic regression analysis revealed that the odds of caseness were significantly lower in participants in the web with tracking condition compared to participants in the control condition (odds ratio 0.16, 95% CI: 0.03 to 0.91, *p* = .039). At 6 month follow up, 43.5% in the web only condition and 50.0% in the web plus tracking condition met clinical caseness compared with 90.9% in the control condition. The odds of meeting caseness criteria were lower in the web with tracking (odds ratio 0.10, 95% CI: 0.02 to 0.56, *p* = .009) and web only (odds ratio 0.08, 95% CI: 0.01 to 0.41, *p* = .003) conditions, compared with the control condition. Post intervention, the number needed to be treated (NNT) under the web only intervention was estimated to be 6.03 (95% CI: 2.72 – ∞ – NNH 27.15) and for the web with tracking intervention 4.43 (95% CI: 2.22–265.75).

Repeating these analyses under the conservative assumption that all participants who discontinued the trial (i.e. failed to complete a post intervention or follow-up questionnaire) retained their last assessed caseness status attenuated effects post intervention. However, 6 month follow-up effects retained, or only narrowly escaped, significance, as the odds of achieving caseness status were lower in participants in the web only (odds ratio 0.17, 95% CI: 0.04 to 0.67, *p* = .010) and web with tracking conditions (odds ratio 0.28, 95% CI: 0.07 to 11.1, *p* = .072), compared to the control condition. This yields NNTs of 6.09 (95% CI: 3.07 – ∞ – NNH 90.15) and 3.63 (95% CI: 2.23–12.29) [33] respectively.

### Dropout and adherence

#### Dropout

Participants who received the web with tracking intervention were less likely to complete a survey at post intervention (odds ratio 0.13, 95% CI: 0.03 to 0.66, *p* = .013), compared with those in the control condition. Neither trial condition nor symptom severity predicted dropout at 6 month follow up.

#### Adherence to the intervention

No significant differences were found in average number of BluePages visits (*t*(81) = 0.388, *p* = .70) or average visit duration (*t*(81) = 0.728, *p* = .47) between participants in the web only and web with tracking conditions, with average number of visits at 2.2 (mean duration 7.4 minutes), and 1.0 (mean duration 3.8 minutes) respectively. Similarly, there was no significant difference in the number of MoodGYM modules completed for the web only and web with tracking conditions (Mann-Whitney *U* = 696.0, *p* = .13). Participants in the web only condition completed an average of 1.5 (SD = 1.89) modules while participants in the web with tracking condition completed an average of 2.0 (SD = 1.88). Approximately one quarter of participants in the web only (15.8%) and web with tracking (17.8%) conditions completed all five modules of the MoodGYM program. 31.6% of participants in the web only and 37.7% of participants in the web with tracking conditions completed 3 or more MoodGYM modules. Half of participants in the web only condition (50.0%) and approximately one third of participants in the web with tracking condition (31.1%) did not complete any modules of the program.

## Discussion

### Summary findings

Web-based CBT, whether delivered with or without tracking, was found to be effective in reducing symptoms of depression both post intervention and at 6 months within a national helpline service. Tracking alone did not differ significantly from the control condition at post intervention or 6 months. At 6 month follow-up, numbers of participants meeting the criteria for clinical caseness on the CES-D were halved in those who received the web intervention compared to those in the control intervention. The effect sizes associated with the web interventions were large, with effects at post intervention and at 6 months equal to those reported for face to face clinician delivered CBT programs for depression [36] and online CBT programs delivered by professionals [37]. The effect sizes exceeded those previously reported for the MoodGYM and BluePages programs [15]. Effects were also larger than any previous reported effect sizes across 11 RCTs comparing Internet interventions to waitlist, treatment as usual or placebo control conditions for depression [6], [15], [17], [38], [39], [40], [41], [42], [43], [44], [45]. This suggests that call centres may be a particularly appropriate setting in which to place evidence-based web programs. The positive results may reflect several factors. Helpline users in particular may prefer to treat themselves, to manage their own health, and to retain a greater sense of control over their treatment. Also, the high baseline depression symptom levels observed in this sample may have enabled greater change in symptoms from pre to post-intervention.

In the context of web delivered programs, weekly telephone support provided by a lay telephone counsellor did not confer any additional advantage in terms of symptom reduction or adherence. Telephone tracking alone was not effective. These findings contribute to the current debate in the e-health literature as to whether guided e-health interventions are more effective or improve adherence [6]. With respect to the latter, we found no evidence that tracking increased program adherence. In fact, somewhat paradoxically, telephone tracking was associated with *decreased* participant retention in the trial from baseline to post intervention for the web with tracking condition. It is possible that weekly contact from the telephone counsellor provided the opportunity for participants in the more demanding web condition to “opt out” of the trial, resulting in higher dropout at the time of the post intervention questionnaire. Telephone contact when not associated with requests to engage with web material (tracking only condition) did not result in increased dropout. It may be that weekly telephone tracking in association with a web intervention was construed as “policing”. There are a number of possible explanations for our findings compared to other online research, reporting better outcomes and adherence for guided interventions for social phobia [46]. Guidance in a study by Titov and colleagues [46] was provided by clinicians who, in contrast to the volunteers in the current study, may have provided clinical input and support. Further, there may be differences in the effects of tracking for depression and social phobia. The content of the tracking call in the current study may also partially account for the results. Unlike other studies, in which telephone support is delivered by a therapist who provides tailored feedback and encouragement, the content of the tracking call in the current study was standardised and telephone counsellors were discouraged from providing any form of personalised counselling during these calls. Finally, the differences may be due, at least in part, to differences in the type of service user. As mentioned previously, the helpline users may prefer greater autonomy in managing their own health.

### Limitations

Intervention completion rates were lower in our study relative to some other trials of web-based treatments for depression [47] but not of other telephone-based services [48]. The reasons for this are unclear. Telephone callers to helplines may experience more stressful events that preclude trial completion. The current trial was a true effectiveness trial employing a volunteer workforce for recruitment and tracking, and hence would be expected to be associated with recruitment and adherence issues relative to the more controlled environment of an efficacy study [49].

Despite the strong effect sizes associated with the intervention, symptom severity remained quite high at post intervention. However, this accords with data on the effectiveness of antidepressants for depression, where overall only about 50% of patients randomised to active treatment were considered improved using intention to treat analyses at post test [50]. Similar rates are reported following face to face CBT [51]. Moreover, the intervention was short, delivered by non-health professionals, and may have constituted the only service received.

Greater dropout in the web with tracking condition may have inflated the effects observed in this condition. It is noteworthy, however, that dropout was not greater in the web only condition, so that the finding of differential dropout from the web with tracking condition does not undermine the primary finding that the web programs were more effective than either tracking alone or the control condition. Two outliers were identified and excluded from the main analysis. Secondary analyses with outliers included confirmed the direction of findings, although we acknowledge that effect sizes were attenuated. A further limitation of the current study was that depression caseness was estimated using a cutoff on the CES-D. Telephone assessment by a health professional using a diagnostic tool such as the Mini-International Neuropsychiatric Interview [52] or the Composite International Diagnostic Interview [53] would have been a better method of determining cases of depression. However, at the start of the trial we were not convinced that participants who normally retain an anonymous, and short but frequent relationship with the helpline service would be willing to undergo extensive psychological testing at baseline. It was of interest, however, that in the context of the current trial, callers were willing to forgo anonymity to receive the intervention.

### Future directions

As far as we can ascertain, the present study is the first to demonstrate that web-based CBT can be effective in the context of a generic, national helpline. The effect sizes were well within the range of those obtained in face to face efficacy trials of CBT delivered by clinicians. In addition, the study demonstrated that a generic helpline could deliver a brief effective intervention within its current service model, and that users of the service were willing to give up their anonymity to participate in the program. The trial demonstrates the potential for individuals in remote or rural locations, those unable to leave home (because of carer responsibilities or mental health symptom severity), and those not wishing to seek traditional medical contact to receive an effective intervention through the telephone and the Internet. At the same time, the trial demonstrates that volunteer counsellors trained to provide “one off” telephone counselling were able to offer a new form of service within their organisation.

From a policy perspective, the volunteer sector was able to provide evidence-based care to individuals with anxiety or depression. Recently, governments in both the United Kingdom and the United States of America have recognised the importance of delivering CBT through general practice by training CBT technicians [54], or through community channels [55]. The current trial offers support for an additional model. Given that it harnesses an existing workforce, this model may well provide a highly cost effective alternative to technician delivered programs in general practice.

The implications for research and service are clear. First, the trial requires replication through other national helplines. Secondly, the replication trial should incorporate either telephone or Internet based diagnosis. In addition, the role of tracking needs to be explored further including the potential role of email contact. There is also a need to find methods for engaging those who dropout before commencing or during the intervention. Critically, given the large number of callers contacting telecounselling services there is a need to ensure that counsellors are prepared to offer the service.

## Supporting Information

Protocol S1 Trial Protocol.
(RTF)

Checklist S1 CONSORT Checklist.
(DOC)
