# Supplementary material for: Internet-Based CBT for Depression with and without Telephone Tracking in a National Helpline: Randomised Controlled Trial
Source: PLoS One. 2011 Nov 30;6(11):e28099. doi: 10.1371/journal.pone.0028099 (PMC3227639; doi:10.1371/journal.pone.0028099)
Supplement: Protocol S1 — Trial Protocol. (RTF) [file pone.0028099.s001.rtf]

ALL APPLICATIONS TO BE TYPED				Version current from 1 February 2004


THE AUSTRALIAN NATIONAL UNIVERSITY

HUMAN RESEARCH ETHICS COMMITTEE

APPLICATION FORM


	Surname of Researcher: Christensen
	First name/s: Helen
	Title (e.g. Ms., Mr., Dr. etc,): Professor	

	Position Held (staff, postgraduate, undergraduate, etc.): Staff	

	Student or Staff ID no. (if applicable): u8804902

	Dept/School/Centre: Centre for Mental Health Research (CMHR)	

	Mailing address: Building 63, Eggleston Road, The Australian National University, Canberra, 
	ACT 0200

	Telephone: 6125 8409
	Fax: 6125 0733
	Email: Helen.Christensen@anu.edu.au

	For students:
	Name of ANU supervisor:
	Email address of ANU supervisor:
	
	PROJECT TITLE: Evaluating the acceptability, feasibility and effectiveness of Internet-based 
	depression interventions as an adjunct to telephone counselling in Lifeline call centres.	

	Date of this application:	01/02/2007
	Anticipated start date for project: 26 March 2007  Anticipated end date: 31 December 2008


1.	The researcher/s
Who are the investigators (including assistants) who will conduct the research and what are their qualification and experience? Please include their Department/School/Centre (or external institution for external researchers). Students should not include supervisors at this point unless they are actually participating in the research project as partner researchers.

Professor Helen Christensen - BA(Hons) (Syd), MPsych, PhD (NSW), FASSA. Professor Christensen is the Director at the CMHR, ANU.  She has particular expertise in the areas of cognitive changes in normal ageing and dementia, individual differences in intelligence, the epidemiology of the common mental disorders, methods to analyse longitudinal data, mental health literacy and the use of the Internet in the prevention of mental disorders.

Associate Professor Kathy Griffiths - BSc(Hons), PhD (ANU). Associate Professor Griffiths is the Director of the Depression and Anxiety Consumer Research Unit at the CMHR, ANU.  She has extensive research experience in the areas of e-mental health (including the development and evaluation of Internet interventions and the development and validation of website quality indicators), self help and help seeking for mental disorders, stigma, mental health literacy and mental health promotion and prevention.  

Professor Andrew Mackinnon - BSc(Hons) (Melb), PhD (Melb). Professor Mackinnon is the Deputy Director at the CMHR, ANU.  Professor Mackinnon is experienced in the quantitative aspects of mental health research. This includes development and analysis of psychometric measures, screening and diagnosis tests, modelling longitudinal data, and the conduct and analysis of controlled trials and interventions in mental health.  

Ms Dawn Smith – MBA (UOW).  Dawn Smith is the CEO of Lifeline Australia and has previously worked with Mental Health Council of Australia and the National Advisory Council on Suicide Prevention.

Mr Trevor Carlyon – BSc, DipPsych, MAppPsych. Mr Carlyon is the Executive Director of Lifeline Community Care Queensland and has a background in clinical psychology.

Ms Nicole Burgess – BSc (Hons) (USQ).  Ms Burgess will be the primary research assistant and trial manager on the project. She has worked as a research assistant at the ANU for the previous 4 years, after successfully completing an Honours thesis investigating the relationship between maternal linguistic style and moral behaviours in young children.  Ms Burgess is based at the Lifeline Queensland office in Brisbane. 

Ms Louise Farrer – BPsych (Hons) (ANU).  This project will form the basis of Ms Farrer's PhD thesis.  She has worked as a research assistant at CMHR since April 2005 and has successfully completed an Honours thesis investigating the relationship between attachment style and optimism in adolescents.


2.	Understanding the national guidelines, the “National Statement on Ethical Conduct in Research Involving Humans” (1999)
Can the proposer certify that the persons listed in the answer to Question 1 above have been fully briefed on appropriate procedures and in particular that they have read and are familiar with the national guidelines issued by the National Health and Medical Research Council (the National Statement on Ethical Conduct in Research Involving Humans) (cited below as the “National Statement”)?  If there are guidelines from any relevant professional body with which the researcher/s are familiar they should also be listed below.

The researchers listed above have read and are familiar with the guidelines issued by the National Health and Medical Research Council.  Ms Farrer and Ms Burgess have completed courses in research methods and ethics during their honours degrees at their respective universities.  They have also read the Australian Psychological Society's code of ethics.


3.	Purpose and design of the proposed research

Purpose
(a) Briefly describe the basic purposes of the research proposed (in plain language intelligible to a non-specialist).

The purpose of this project is to investigate the use of evidence-based Internet therapy interventions for depression in Lifeline call centres. The project aims to compare the effectiveness of cognitive behavioural therapy and depression information (accessed online) in addition to telephone counselling with telephone counselling alone for treating depression.  This research is being conducted in partnership with Lifeline Australia (funded through an Australian Research Council Linkage Grant).

Despite the popularity of the web, little is known about the capacity of the Internet to improve knowledge and promote positive health.  The project aims to establish whether the use of Internet technology is superior to traditional methods for delivering health information. It addresses Lifeline's current mission to transform its health service through the provision of evidence-based information and preventative tools to callers.  The research will also be aimed at the significant proportion of callers who use Lifeline on a recurrent and chronic basis for help with emotional problems, but who are not necessarily in crisis.  If successful, this project could result in greater numbers of Australians with mental health problems receiving appropriate evidence-based care.

A scoping phase has been carried out to gain more accurate information about the mental health of Lifeline callers and their willingness to participate in evidence-based research (Protocol 2005/269).  The data we have collected indicate that a significant number of callers would be willing to participate in the research and provide personal information to do so.  Moreover, the levels of depression and anxiety symptoms in this sample suggest that a service targeting these problems could be of benefit.

Design
(b) Outline the design of the project (in plain language intelligible to a non-specialist).  (If interviewing people or administering a survey/questionnaire, please attach either a list of the broad questions you propose to ask, or a copy of the questionnaire.)

Trial design and flow

The project will employ a randomised controlled trial to evaluate the effectiveness of 2 evidence-based depression websites: MoodGYM (online cognitive behaviour therapy) and BluePages (online depression information) with callers to Lifeline's Brisbane call centre.  At the conclusion of or during each counselling call, telephone counsellors will invite callers to receive further information about the project (for further details see Section 6).

Callers recruited from the initial telephone counsellor invitation will receive a call-back from a project staff member to discuss the project in more detail. Callers will be informed about the purpose of the trial and what participation will involve (including voluntary participation, randomisation to a trial condition, confidentiality, and time requirements). Callers will also be asked a series of screening questions to assess their eligibility for participation in the trial.  The inclusion and exclusion criteria for the study are as follows:
Inclusion criteria:
· English speaking
· Internet access
· Age 18 or older
· Elevated depression and anxiety symptoms (assessed using cut-off of 22 or above   on the Kessler Psychological Distress Scale)
· Willingness to participate

Exclusion criteria:
· History of psychosis, schizophrenia or bipolar disorder
· Currently receiving CBT
· Reading impairment
Ineligible participants will be offered health information and materials relating to the BluePages and MoodGYM websites, although they will not be part of the trial.

Participants eligible and willing to participate in the project will be posted an information pack that contains a project information flyer to keep, a consent form and a pre-intervention questionnaire. Participants will be asked to sign the consent form and return it with the completed pre-intervention questionnaire in a reply-paid envelope as soon as possible.

As soon as the consent form and pre-intervention questionnaire have been returned, participants will be randomised to one of the following four conditions:
Program (1): Weekly visits to BluePages and MoodGYM over 6 weeks (Internet only).
Program (2): Weekly visits to BluePages and MoodGYM over 6 weeks plus weekly contact from a telephone counsellor (Internet plus tracking).
Program (3): Weekly contact from a telephone counsellor over 6 weeks (Tracking only).
Program (4): Treatment as usual (Control).  Treatment as usual will consist of usual caller-initiated contact with Lifeline's crisis counselling service.

	Program 1	Program 2	Program 3	Program 4	
Week 0	Initial contact from telephone counsellor	Initial contact from telephone counsellor	Initial contact from telephone counsellor	Initial contact from telephone counsellor	
Week 1	BluePages	BluePages + contact from telephone counsellor	Contact from telephone counsellor	Treatment as usual	
Week 2	MoodGYM Module 1	MoodGYM Module 1 + contact from telephone counsellor	Contact from telephone counsellor	Treatment as usual	
Week 3	MoodGYM Module 2	MoodGYM Module 2 + contact from telephone counsellor	Contact from telephone counsellor	Treatment as usual	
Week 4	MoodGYM Module 3	MoodGYM Module 3 + contact from telephone counsellor	Contact from telephone counsellor	Treatment as usual	
Week 5	MoodGYM Module 4	MoodGYM Module 4 + contact from telephone counsellor	Contact from telephone counsellor	Treatment as usual	
Week 6	MoodGYM Module 5 + wrap-up from telephone counsellor	MoodGYM Module 5 + wrap up from telephone counsellor	Wrap up from telephone counsellor	Wrap up from telephone counsellor	

Weekly telephone counsellor contact will consist of a 10 minute phone call discussing the relevant sections of the Internet program visited during each week (for Program 2) and various lifestyle issues such as activity level, nutrition, career and health issues (for Program 3).   Following the intervention period, participants will complete a post-intervention questionnaire, and will be asked to complete follow-up questionnaires at 6 and 12 months (Note: 6 and 12 month follow-up questionnaires are the same).  Participants who receive Program 4 (the control condition) will be wait-listed to receive the Program 1 (Internet only condition) six months after their intervention phase is complete.

Questionnaires measures

Copies of pre, post, follow-up questionnaires are attached.  The primary outcomes of interest are depression and anxiety symptomology.  Depression will be measured using the Centre for Epidemiologic Studies Depression Scale (CES-D), which is a well-established, valid and reliable scale for identifying depression in the general population.  Anxiety will be measured using the Anxiety subscale from the Depression Anxiety and Stress Scales, which was developed and validated with an Australian population sample.  Secondary outcome measures include:
Outcome	Scale	# of items	Pre	Post	6 m	12 m	
Depression & anxiety history	Depression and anxiety screening items from the Prime-MD.  Other items developed by the researchers.	6	√				
Dysfunctional thoughts	Automatic Thoughts Questionnaire	8	√	√	√	√	
Personal stigma	Depression Stigma Scale 	9	√	√	√	√	
Beliefs about Internet	Developed by researchers	2	√	√	√	√	
Mental Health Literacy	Developed by researchers	33	√	√	√	√	
Helpseeking	Developed by researchers	34	√	√	√	√	
Depression literacy	Developed by researchers	11	√	√	√	√	
CBT literacy	Developed by researchers	10	√	√	√	√	
Internet program usefulness/usage	Developed by researchers	14		√	√	√	
Condition preference	Developed by researchers	1	√				
Alcohol use	Alcohol Use Disorders Identification Test (AUDIT)	5	√	√	√	√	
Quality of life	EURO-HIS 8	8	√	√	√	√	
Disablement	2 items from the Medical Outcomes Study Short Form-12 (SF-12)	2	√	√	√	√	
Suicidal ideation	4 items from the General Health Questionnaire-28 (GHQ-28)	4	√	√	√	√	
Lifeline use	Developed by the researchers	1	√	√	√	√	
Demographics	Age, sex, marital status, education level, employment status, current study.	11	√				


4. Sources of data involving humans
To ensure compliance with privacy legislation the committee needs to know your sources of information, i.e. where you are obtaining data involving humans. If you are using individual participants, tick at (a). If you are accessing personal records held by government departments or agencies, or by other bodies, e.g. private sector organisations, please tick and complete the relevant sections (b), (c) and/or (d) below.

(a) Individual subjects						(√)
	(b) Commonwealth Department/s or agency (specify)*	(  )……………………….
	(c) State/Territory Department/s or agency (specify*		(  )……………………….
	(d) Other sources (specify)					(  )….…………………….
*Please include an estimate of how many records you expect to access:……………………….


5.	Personal identifiable data for medical/health research
Are you obtaining personal identifiable data specifically for medical/health research that is held by a government or private sector agency? (The committee needs this information to determine whether it needs to comply with relevant National Health and Medical Research Council guidelines relating to privacy legislation.)


NO


6.	Recruitment
Describe how participants will be recruited for this project.  Indicate how many participants are likely to be involved, how initial contact will be made, and how participants will be invited to take part in this project.  A copy of any relevant correspondence should be attached to this application. Does the recruitment process raise any privacy issues, e.g. does the researcher plan to access personal information to identify potential participants without their knowledge or consent?  Describe the steps to be taken to ensure that participation or refusal to participate will not impair any existing relationship between participants and researcher or institution involved.

Callers to the Lifeline Brisbane call centre will be recruited into the trial by telephone counsellors, at the end of the counselling call (or if an appropriate time arises during the call).  Counsellors will exclude callers where it is deemed inappropriate to invite them to participate (i.e. when the major focus of the call has been suicide/crisis and/or the caller is highly distressed).  Telephone counsellors will introduce callers to the project and invite them to find out more information. Callers who express interest in finding out more information about the research will be asked if they have access to the Internet and those with Internet access will then be asked to provide their name and phone number in order for a project staff member to call them back (in the next few days) to discuss the project further.  Based on call rates, we expect an average of 20-25 callers per day to be initially approached to participate in the project.

Telephone counsellors will record interested callers' names and telephone numbers using a secure online password-protected program (APOLLO).  Telephone counsellors will inform callers that their involvement in the study is voluntary and if they decline the invitation to participate they will be encouraged to call Lifeline again at any time.  Following recruitment and screening, we aim to have a total sample size of 400 participants (100 per condition).


7.	Arrangements for access to identifiable data held by another party
In cases where participants are identified from information held by another party (e.g. government department, non-governmental organisation, private company, community association, doctor, hospital) describe the arrangement whereby you will gain access to this information.  Attach any relevant correspondence.

Not applicable.


8.	Vulnerable participants
Will participants include students, children, the mentally ill or others in a dependent relationship? If so, provide details.

Yes. The aim of the research is to investigate the effectiveness of Internet interventions in lowering depression symptoms in Lifeline callers.  All participants will be adults, however, eligible participants will be those who have elevated symptoms of depression or anxiety.  Participants are also likely to be experiencing personal or emotional difficulties as they have called Lifeline to discuss their problems with a counsellor. Lifeline counsellors will assure participants that their involvement in the study is voluntary, and that their choice not to participate does and will not affect any current or future assistance they may receive from Lifeline. Lifeline telephone counsellors have been trained in responding to emotional reactions from callers and will be provided with training for recruiting and contacting participants during their involvement in the study.


9.	Payment
Will payment be made to any participants?  If so, give details of arrangements.

No


10.	Consent
Describe the consent issues involved in this proposal (see the National Statement, in particular Section 1.7-12, and other sections relevant to your research). Describe the procedures to be followed in obtaining the informed consent of participants and/or of others responsible. Attach any relevant documents such as a consent form, information sheet, letter of invitation etc.  If you do not propose to obtain written consent (e.g. if working with non-literate people) give a detailed explanation of the reasons for seeking oral consent, describe the procedure you intend to adopt, and specify the information to be provided to participants.  If you have answered YES to Question 8 above please address any issues of consent and the possibility of coercion.

Once participants have been screened for eligibility they will receive a consent form and information sheet by post.  The consent form contains a brief description of the project, information about random assignment to the trial conditions, amount of time participation will involve, confidentiality, and contact information for the researchers and the ANU Human Ethics Officer.  The information sheet is in the form of a flyer for the participant to keep.  It contains the same information outlined in the consent form.

Given the potentially dependent relationship between callers and telephone counsellors and research staff, it will be stressed to callers throughout the project (recruitment, consent, pre, post and follow-up assessments) that their participation is entirely voluntary and that they may withdraw from the study at any time without penalty.


11.	Protection of privacy (confidentiality)
Describe the confidentiality issues involving in this proposal. Give details of the measures that will be adopted to protect confidential information about participants, both in handling and storing raw research data and in any publications. Blanket guarantees of confidentiality are not helpful. If the term “confidential” is used in information provided to participants, a full description of what precisely confidentiality means in the context of this research should be given. You should be aware that, under Australian law, any data you collect can potentially be subpoenaed. Depending on the nature of your research, it may be helpful to qualify promises of confidentiality with terms such as “as far as possible” or “as far as the law allows”.  [See the National Statement, in particular Sections 1.19, 18 and Appendix II]

It is outlined to participants in the consent form and information flyer that the personal information collected during the project may be of a personal nature and will be identified by an ID number only.  All files linking participant names and other personal information to ID numbers will be stored separately from each other and from raw research data.  Once completed consent forms have been received, the signed section will be detached from the rest of the form (that contains the participant's ID number) and stored separately.  Completed surveys will be stored in a locked filing cabinet at the CMHR, and all other information (including entered data, website data, and information collected over the phone) will be recorded and stored on password protected computers at Lifeline's Brisbane office and the CMHR.  These files will only be accessed by the researchers.


12.	Cultural or social considerations
Comment on any cultural or social considerations that may affect the design of the research. [See the National Statement, in particular Sections 1.2 and 1.19].

We do not forsee any cultural or social considerations affecting the design of this research.


13.	How the research might impact on participants
Describe and discuss any possible impact of the proposed research on the participants or their communities that you can foresee. This might include psychological, health, social, economic or political changes or ramifications. Discuss how you will try to minimise any impact.  [See the National Statement, in particular Sections 1.3 to 1.6 and Section 1.14]

We do not foresee any negative health, social, economic or political changes or ramifications as a result of this research. It is likely that this research will have a positive effect and result in greater numbers of Australians with mental health problems receiving evidence-based interventions for depression, including areas where such interventions are currently largely inaccessible.

It is possible that some Lifeline callers may experience distress due to their participation in the research, as portions of the subject matter of the interventions and questionnaire items are of a sensitive nature.  Most of the interaction involving participants in the project will be handled by telephone counsellors, who in addition to their thorough counselling training and experience, will be trained in all aspects of the trial.  All Lifeline counsellors in the Brisbane call centre will be aware that the research project is taking place and that they may receive calls about participation in the project.  If a caller becomes distressed at any stage during their participation in the study they will be encouraged to contact Lifeline's crisis line and/or the researchers directly.  Participants will be made aware that their involvement in the study is in no way connected to the help that have previously and continue to receive from Lifeline's usual crisis counselling service, and that they are free to continue to use this service as they normally would throughout their involvement in the study.  One of the questionnaire measures relates to suicidal ideation.  Participants are encouraged in the information flyer to contact Lifeline if they are feeling suicidal, and given that they have previously engaged with Lifeline, we believe they will have a propensity for this.  All Lifeline telephone counsellors are trained and experienced in the use of the ASIST suicide intervention model.


14.	Other ethical and any legal considerations
Comment on any other ethical considerations that are involved in this proposal, including any potential for legal difficulties to arise for participants.


No other ethical or legal considerations are forseen.


15.	Benefits versus risks
Describe the possible benefit/s to be gained from the proposed research. Explain why these benefits outweigh or justify any possible discomforts and risks to participants. In framing your explanation make explicit reference to the ethical considerations mentioned in your answers to previous questions on this form. [See the National Statement, in particular Sections 1.3-6 and 1.13-14]

Depression is a major public health concern in Australia, however many people do not seek or receive adequate help.  The health care environment is changing radically with the development of technologies and this project directly investigates how new technologies can be used to deliver much needed high-quality health information and training to the community.  This will be the first trial evaluating the use of web applications in the delivery of depression interventions to users of telephone counselling centres. There have been no randomized controlled trials of either web or telephone health services either in Australia or internationally.  This trial has immediate practical importance given Lifeline's growing mental health consumer base and commitment to integrate Internet technologies into the delivery of its counselling and crisis intervention service.  Positive outcomes from this trial will result in greater numbers of Australians with mental health problems receiving evidence-based care.

At an academic level, the trial addresses the important issue of whether web applications are as efficacious as traditional methods of health service delivery, and whether supervision and tracking are important for outcomes to be delivered – these outcomes include learning, health behaviour and symptom change, adherence and dropout. The aim of this study is to generate information that is directly applicable to a broad range of web applications in areas which seek to engage, inform and interact with participants and which, by doing so, produce change in behaviour, emotional state or knowledge in users. This is relevant to areas beyond the mental health sector including education, professional training and management.


16.	Handling possible problems arising from the research
Describe the arrangements you have made to handle concerns and complaints by participants, or emergencies involving participants or researchers.

Participants will be provided with the contact details of the project researchers and the ANU Human Ethics Officer if they wish to discuss any aspect of the project or have any concerns or complaints.  Participants will also be encouraged to use Lifeline's crisis counselling service in the event that participation in the project causes them any distress.  Participants involved in the Internet programs will be provided technical assistance through a project e-mail address to help them access and navigate the websites.


RESEARCH PROTOCOL CHECKLIST

There are some key ethical principles that need to be addressed in your protocol (as an ethics application is known). In particular the committee needs to see how you have addressed the issue of informed consent and the issue of confidentiality, i.e. how the identities of participants will be protected in the raw research data and in published material. The usual way to obtain informed consent is in writing, by use of a consent form that is signed by the participant and retained by you. Because you retain the consent form the same information needs to be included in an information sheet that participants retain. Both the consent form and the information sheet should include your name, contact details, title and brief description of the project, details on how the identities of participants will be protected (both when storing the raw research data and in its published form), a statement that participation is voluntary and participants can withdraw at any time, and contact details for the Human Research Ethics Committee in case of any ethical concerns. If you do not propose to seek written consent, you need to explain why oral consent will be sufficient and how you propose to obtain it.  


Please tick the relevant boxes below to indicate what has been included in your protocol:

Outline of proposal and purpose							Yes  X		No  
Measures to be taken to protect confidentiality					Yes  X		No  
Explanation of how written informed consent will be obtained				Yes  X		No  

If written consent is not being sought, justification of a verbal consent procedure is included        Yes  

Full details on investigators (name, institution, etc.) 					Yes  X		No  
All researchers on this project are familiar with the national guidelines (National Statement) 
		Yes  X		No  
Details re how participants will be recruited						Yes  X		No  

Is personal data from a Commonwealth department/agency or private sector organisation being used? 												Yes  		No  X
Details on how cultural and social sensitivities will be addressed			Yes  X		No  

Consideration of likely risk to participants (e.g. psychological stress; cultural, social, political or economic ramifications) 									Yes  X		No  

Do your research participants include:
Aboriginal or Torres Strait Islander peoples			Yes  		No  X
Children and young people (i.e. minors under the age of 18) 	Yes  		No  X
People with an intellectual or mental impairment		Yes  X		No  
People highly dependent on medical case			Yes  		No  X
People in dependent or unequal relationships			Yes  X		No  

Do you intend to pay participants? 						Yes  		No  X

Description of method and amount is included 	Yes  

Description of clinical facilities (for medical research) 			Yes  		No  X

Period of research							Yes  X		No  
